# PreAIP: Computational Prediction of Anti-inflammatory Peptides by Integrating Multiple Complementary Features

**DOI:** 10.3389/fgene.2019.00129

**Published:** 2019-03-05

**Authors:** Mst. Shamima Khatun, Md. Mehedi Hasan, Hiroyuki Kurata

**Affiliations:** ^1^Department of Bioscience and Bioinformatics, Kyushu Institute of Technology, Fukuoka, Japan; ^2^Biomedical Informatics R&D Center, Kyushu Institute of Technology, Fukuoka, Japan

**Keywords:** inflammatory disease, anti-inflammatory peptides prediction, feature encoding, feature selection, random forest

## Abstract

Numerous inflammatory diseases and autoimmune disorders by therapeutic peptides have received substantial consideration; however, the exploration of anti-inflammatory peptides via biological experiments is often a time-consuming and expensive task. The development of novel *in silico* predictors is desired to classify potential anti-inflammatory peptides prior to *in vitro* investigation. Herein, an accurate predictor, called PreAIP (Predictor of Anti-Inflammatory Peptides) was developed by integrating multiple complementary features. We systematically investigated different types of features including primary sequence, evolutionary and structural information through a random forest classifier. The final PreAIP model achieved an AUC value of 0.833 in the training dataset via 10-fold cross-validation test, which was better than that of existing models. Moreover, we assessed the performance of the PreAIP with an AUC value of 0.840 on a test dataset to demonstrate that the proposed method outperformed the two existing methods. These results indicated that the PreAIP is an accurate predictor for identifying AIPs and contributes to the development of AIPs therapeutics and biomedical research. The curated datasets and the PreAIP are freely available at http://kurata14.bio.kyutech.ac.jp/PreAIP/.

## Introduction

Inflammation responses occur under the normal conditions when tissues are damaged by bacteria, toxins, trauma, heat, or any other reason (Ferrero-Miliani et al., 2007). These responses cause chronic autoimmune and inflammation disorders, including neurodegenerative disease, asthma, psoriasis, cancer, rheumatoid arthritis, diabetes, and multiple sclerosis (Zouki et al., 2000; Steinman et al., 2012; Tabas and Glass, 2013; Patterson et al., 2014; Hernández-Flórez and Valor, 2016). Numerous inflammation mechanisms are crucial for the upkeep of the state of tolerance (Miele et al., 1988; Corrigan et al., 2015). Numerous endogenous peptides recognized through inflammatory reactions function as anti-inflammatory agents can be employed by new therapies for autoimmune and inflammatory illnesses (Gonzalez-Rey et al., 2007; Delgado and Ganea, 2008). The immunotherapeutic aptitude of these anti-inflammatory peptides (AIPs) has various clinical applications such as generation of regulatory T cells and inhibition of antigen-specific T(H)1-driven responses (Delgado and Ganea, 2008). Moreover, certain synthetic AIPs act as effective therapeutic agents for autoimmune and inflammatory disorders (Zhao et al., 2016). For instance, chronic adenoidal direction of human amyloid-β peptide causes an Alzheimer's disease. Mice models result in compact deposition of amyloid-β peptides, which is a pathological marker of Alzheimer's disease, astrocytosis, microgliosis, and neuritic dystrophy in the brain (Boismenu et al., 2002; Gonzalez et al., 2005; Kempuraj et al., 2017). The present therapy for autoimmune and inflammatory disorders involves the use of non-specific anti-inflammatory drugs and other immunosuppressants, which are frequently related to different side effects, such as initiation of a higher possibility of infectious diseases and ineffectiveness alongside inflammatory disorders (Tabas and Glass, 2013).

Notwithstanding the increasing number of experimentally examined AIPs *in vivo*, the molecular mechanism of AIP specificity remains largely unknown. On the other hand, large-scale experimental analysis of AIPs is time-consuming, laborious, and expensive. An alternative, computational approach that provides an accurate and reliable prediction of AIPs is required to complement the experimental efforts and to access the prompt identification of potential AIPs prior to their synthesis. To date, two *in silico* methods have been proposed to predict AIPs (Gupta et al., 2017; Manavalan et al., 2018). In 2017 Gupta et al. employed hybrid features with a support vector machine (SVM) classifier to develop the AntiInflam predictor (Gupta et al., 2017). Manavalan et al. developed the AlPpred predictor by using the primary sequence encoding features with a random forest (RF) classifier (Manavalan et al., 2018). These two methods used the primary sequence feature information without considering any evolutionary or structural features.

Nonetheless, the performance of the abovementioned existing predictors is not sufficient and remains to be improved. In this study, we have developed an accurate predictor named PreAIP (Predictor of Anti-Inflammatory Peptides) by integrating multiple complementary. We investigated different types sequence features including the primary sequence, evolutionary, and structural through a RF classifier. The PreAIP achieved higher performance on both the training and test datasets than the existing methods. In addition, we obtained valuable insights into the essential sequence patterns of AIPs.

## Materials and Methods

### Dataset Collection

To construct the PreAIP, we collected training and test datasets from a recently published article of the AIPpred (Manavalan et al., 2018) and the IEDB database (Vita et al., 2019). A peptide was considered as anti-inflammatory (positive sample) if the anti-inflammatory cytokines of peptides induce any one of IL-10, IL-4, IL-13, IL-22, TGFb, and IFN-a/b in T-cell analyses of mouse and human (Marie et al., 1996; Jin et al., 2014). Meanwhile, the linear peptides for anti-inflammatory cytokines were considered non-AIPs (i.e., negative samples). To solve the overfitting problem of the prediction model, CD-HIT was employed with a sequence identity threshold of 0.8 (Huang et al., 2010). After eliminating redundant peptides, the same training and test samples were retrieved from the AIPpred predictor (Manavalan et al., 2018). More reliable performance would be achieved by using a more stringent criterion of 0.3 or 0.4, as executed in (Hasan et al., 2016, 2017a). However, this study did not use such a stringent criterion, because the length of the currently available AIPs is between 4 and 25. If we apply a stringent criterion of <0.8, the number of the available AIPs is greatly reduced so that we cannot retrieve the datasets employed by the previous predictor (Manavalan et al., 2018). The collected training dataset results in 1,258 positive and 1,887 negative samples, and the test dataset contains 420 positive and 629 negative samples. All of curated datasets are included in our web server.

### Computational Framework

**Figure 1:**
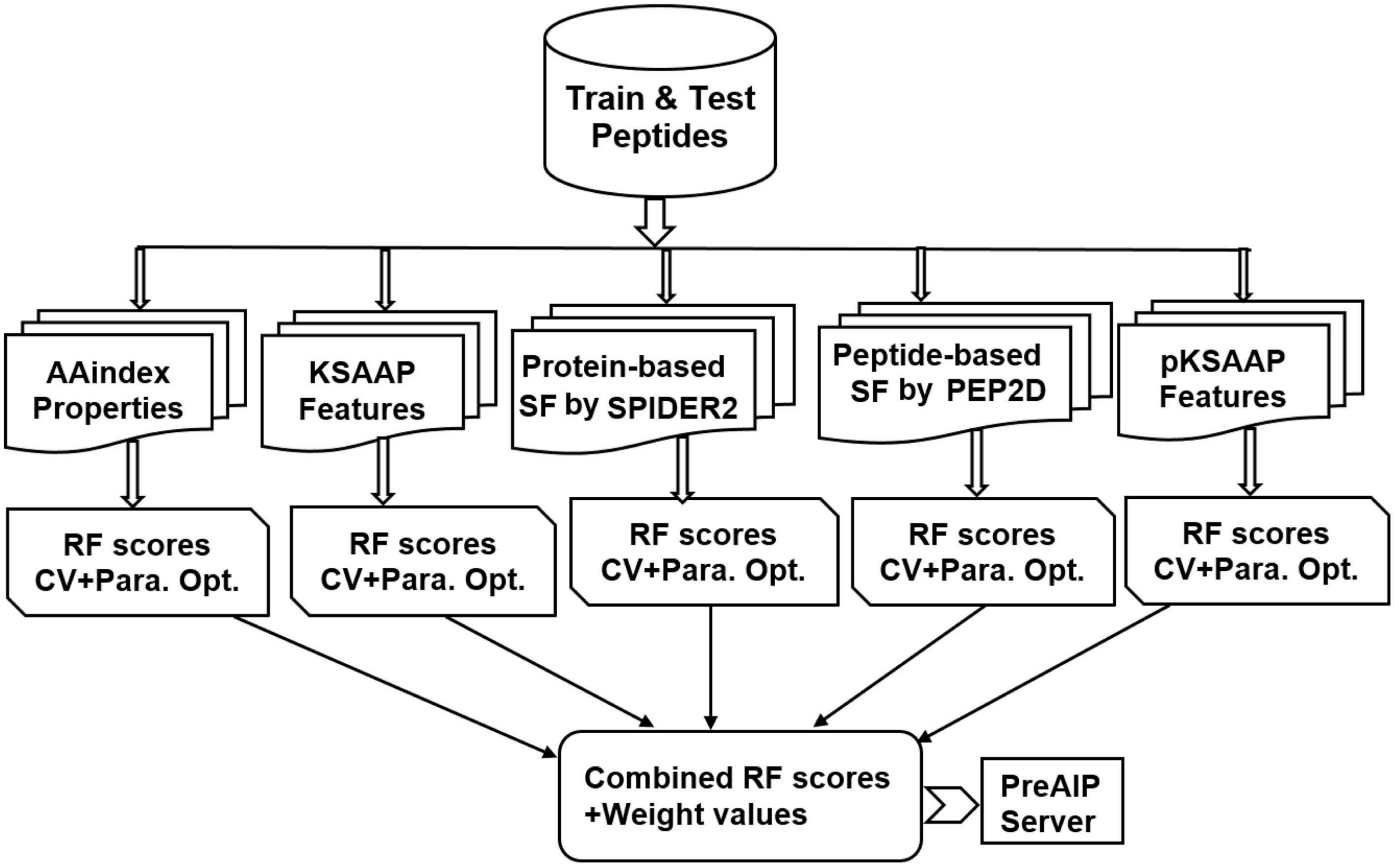
Computational framework of PreAIP.

An overall computational framework of the proposed PreAIP is shown in Figure 1. After collecting the positive and negative AIPs from the AIPpred server (Manavalan et al., 2018), their sequence datasets were transformed into the primary sequence, evolutionary and structural features. We considered polypeptides with 1 to 25 natural amino acids. When the peptide contains less than 25 residues, our scheme provides gaps (-) to the missing residues to compensate a peptide length of 25. To encode the primary sequence features, we employed two encoding methods of the composition of *k*-spaced amino acid pairs (KSAAP) and AAindex properties. An evolutionary feature was encoded by using the position specific encoding matrix, i.e., profile-based composition *k*-space of amino acid pair (pKSAAP). The structural feature (SF) was encoded by using SPIDER2 (Yang et al., 2017) and PEP2D (http://crdd.osdd.net/raghava/pep2d/) bioinformatics tools. The resulting five types of descriptors were independently put into RF models to produce five consecutive, independent RF prediction scores. Those RF scores were linearly combined using the weight coefficients to obtain the final prediction score. A web server was developed to implement the PreAIP.

### Feature Encoding

The PreAIP was constructed based on a binary classification problem (positive AIPs and negative-AIPs) through RF algorithms. The extraction of a set of relevant features is a crucial step to present a classifier. To keep the generated feature vectors, a high-quality peptide encoding method is necessary. As a substitute of the simple binary representation, we adopted five types of complicated feature encoding methods: AAindex, KSAAP, SPIDER2, PEP2D, and pKSAAP, which are briefly described in the following subsections.

### Amino Acid Index Properties

Numerical physicochemical properties of amino acids exist in the AAindex database (version 9.1) (Kawashima et al., 2008). After assessing different types of AAindex indices, we selected 8 types of high indices (HI) and ordered them from HI1 to HI8 (Table S1). In a peptide sequence with length *L*, a (*L* × 20) feature vector was generated through the AAindex encoding.

### KSAAP Encoding

The KSAAP encoding descriptor is widely used in bioinformatics research (Carugo, 2013; Hasan et al., 2018a,b). The procedure of KSAAP is briefly described as follows. Peptide sequences contain (20 × 20) types of amino acid pairs (i.e., AA, AC, AD, …, YY)_400_ for every single *k*, where *k* denotes the space between two amino acids. The optimal *k*_*max*_ was set to 0–4 to generate (20 × 20 × 5) = 2,000 dimensional feature vectors for each corresponding peptide sequence. Details of the KSAAP encoding method are described elsewhere (Hasan et al., 2015).

### Structural Features

#### Protein-Based SF

The protein-based SF features are generated by the SPIDER2 software that is widely used in bioinformatics research (Yang et al., 2017; López et al., 2018). Three types of features were generated by SPIDER2: accessible surface area (ASA), backbone torsion angles (BTA), and secondary structure (SS). The BTA generated 4-type feature vectors of phi, psi, theta and tau. The SS generated 3-type feature vectors of helix, strand and coil. Totally, 8-type feature vectors were generated SPIDER2. For each peptide sequence, (*L* × 8) dimensional feature vectors were generated, where *L* was the length of a given AIP.

#### Peptide-Based SF

We employed PEP2D to generate a peptide structure prediction feature (http://crdd.osdd.net/raghava/pep2d/). The PEP2D generated three types of probability scores: Helix Prob, Sheet Prob, and Coil Prob. For each peptide sequence, (*L* × 3) dimensional feature vectors were generated, where *L* was the length of a given AIP.

### pKSAAP Encoding

In protein or peptide sequence analysis, the PSSM provides useful evolutionary information. This matrix measures the replacement probability of each residue in a protein with all the residues of the genomic code. The *PSSM profile* was created by using PSI-BLAST (version of 2.2.26+) against the whole Swiss-Prot NR90 database (version of December 2010) with two default parameters, an e-value cutoff of 1.0 × 10^−4^ and an iteration number of 3 (Hasan et al., 2015). Then, we extracted the feature vectors using the given peptide sequences. After generating the PSSM profile, we generated possible *k*-space pair composition from the PSSM, i.e., pKSAAP, in the same manner as the previous study of protein pupylation site prediction (Hasan et al., 2015). When an optimal *k*-space was between 0 and 4, a (5 × 20 × 20 = 2,000) dimensional feature vector was generated.

Moreover, we utilized a similarity-search-based tool of BLAST (version of ncbi-blast-2.2.25+) (Altschul et al., 1997; Bhasin and Raghava, 2004) to investigate whether a query peptide belongs to AIPs or not. The BLASTP with an e-value of 1.0 × 10^−2^ was used for the whole Swiss-Prot NR90 database (version of December 2010).

### Feature Selection

To find the top ranking features for predicting AIPs, a well-established, supervised method for feature dimensionality reduction, Information Gain (IG) (Azhagusundari and Thanamani, 2013; Huang, 2015; Manavalan et al., 2018), was used through a WEKA package (Frank et al., 2004). A large value of the IG indicates that the corresponding residues have a great impact on prediction performance. The IG processes the decrease in entropy when given information is used to group values of an alternative (class) feature. The entropy of feature *U* is defined as

(1) $$H\left(U\right)=-\sum_{i}P(u_{i})log_{2}\left(P\left(u_{i}\right)\right)$$

where *u*_*i*_ is a set of values of *U* and *P* (*u*_*i*_) is the prior probability of *u*_*i*_. Conditional entropy *H*(*U/V*), given another feature *V*, is defined as

(2) $$H\left(U|V\right)=-\sum_{j}P\left(v_{j}\right)\sum_{i}P\left(u_{i}|v_{j}\right)log_{2}(P(u_{i}|v_{j}))$$

where *P* (*u*_*i*_ | *v*_*j*_) is the posterior probability of *U* given by the value *v*_*j*_of *V*. The *IG* is defined as the decreased entropy calculated by subtracting the conditional entropy of *U* given by *V* from the entropy of *U*, as follows.

(3) IG(U|V)=H(U)−H(U|V)

### Random Forest

The RF is a supervised machine learning algorithm (Breiman, 2001) and is widely used for various biological problems (Manavalan et al., 2017, 2018; Bhadra et al., 2018; Hasan and Kurata, 2018). In brief, the following steps are carried to construct *n* trees of the RF model. Initially, to obtain a new dataset, *N* samples are obtained from the training set by random selection with replacement procedures. To get *n* different datasets this procedure is repeated *n* times and *n* decision trees are built based on the *n* datasets. In this assembling process, for *K* input features, *k* (*k* << *K*) features are selected randomly, where *k* is the constant during construction of the RF. To split the node, a *gini* impurity criterion is used from the given features. To grow completely, each decision tree is grown without pruning. Afterward getting *n* decision trees, the class with the most votes is the final prediction (Breiman, 2001). An R package was implemented to train the proposed model (https://cran.r-project.org/web/packages/randomForest/). We set *n* to 1000 through the 10-fold cross-validation (CV) test, which is large enough to gain stable prediction.

### Other Machine Learning Algorithms

The performance of the RF was characterized in comparison to three commonly used machine learning algorithms: Naive Bayes (NB) (Lowd, 2005), SVM (Hearst, 1998), and artificial neural network (ANN) (Michalski et al., 2013). We used the NB and ANN algorithms of the WEKA software (Frank et al., 2004) and the SVM algorithm with a kernel radial basis function (RBF) of the LIBSVM package (https://www.csie.ntu.edu.tw/~cjlin/libsvm/). In the NB algorithm, we set batch size to 1,000 through the 10-fold CV via the WEKA software. For the ANN algorithm, we considered “MultilayerPerceptron –L 0.3 –M 0.2 –N 500 –V 0 S 0 –E 20 –H a” via the WEKA software. To optimize the parameters of the SVM model, the cost and gamma functions were set to 8 and 0.03125 for KSAAP, respectively, via the LIBSVM package. Similarly, the cost and gamma functions were set to 2 and 0.0123 for AAindex, 32 and 0.0625 for pKSAAP, 16 and 0.125 for SPIDER2, and 8 and 0.015625 for PEP2D.

### Combined Method

To make an efficient and robust prediction model, optimization of incorporative feature methods is generally essential. We linearly combined the RF scores of the five encoding methods: AAindex, KSAAP, SPIDER2, PEP2D, and pKSAAP, using the following formula (Hasan et al., 2017b):

(4) $$\begin{array}{l} \text{Combined}=w_{1}\times \text{SPIDER}2+w_{2}\times \text{PEP}2\text{D}+w_{3}\times \text{KSAAP}\\ \;+w_{4}\times \text{AAindex}+w_{5}\times \text{pKSAAP} \end{array}$$

where *w*_1_*, w*_2_*, w*_3_*, w*_4_, and *w*_5_ are the weight coefficients indicating the strength of the five descriptors; the sum of *w*_1_, *w*_2_, *w*_3_*, w*_4_, and *w*_5_ is 1. We adjusted each weight from 0 to 1 with an interval of 0.05. When *w*_1_, *w*_2_, *w*_3_*, w*_4_, and *w*_5_ were 0.00, 0.00, 0.15, 0.25, and 0.6, respectively, the AUC value on the CV of training dataset was maximal. Therefore, the linear combination of the three successive RF models of KSAAP, AAindex, and pKSAAP was actually “Combined.”

### Performance Assessment

To investigate the performance of the PreAIP, the threshold-dependent and threshold-independent indices were measured. Using the threshold-dependent indices, four widely used statistical measures denoted as accuracy (Ac) specificity (Sp), sensitivity (Sn), and Matthews correlation coefficient (MCC), respectively, were considered. The four outcomes are presented in the following formulas,

(5) $$\text{Ac}=\frac{\text{TP}+\text{TN}}{\text{TP}+\text{FP}+\text{TN}+\text{FN}}$$

(6) $$\text{Sn}=\frac{\text{TP}}{\text{TP}+\text{FN}}$$

(7) $$\text{Sp}=\frac{\text{TN}}{\text{TN}+\text{FP}}$$

(8) $$\text{MCC}=\frac{\left(\text{TP}\times \text{TN})-(\text{FP}\times \text{FN}\right)}{\sqrt{\left(\text{TN}+\text{FN})\times (\text{TP}+\text{FP})\times (\text{TN}+\text{FP})\times (\text{TP}+\text{FN}\right)}}$$

where TP exemplifies the number of correctly predicted positive samples; TN the number of correctly predicted negative samples; FP the number of incorrectly predicted positive samples, and FN the number of incorrectly predicted negative samples. Furthermore, we used the receiver operating characteristics (ROC) curve (Sn vs. 1-Sp plot) to evaluate the area under the ROC curve (AUC) of the threshold-independent parameter (Centor, 1991; Gribskov and Robinson, 1996).

Since the balance between the correctly predicted AIPs and non-AIPs is critically responsible for accurate prediction, Sp and Sn are intuitive, intelligible measures. Typically, high Sp decreases Sn. In this study, the prediction performance of the PreAIP for the training dataset was evaluated with a stepwise change in Sp. We calculated Sn, Ac, and MCC at high (0.903), moderate (0.801) and low (0.709) levels of Sp. These three levels of Sp were given by setting the high (0.468), moderate (0.388), and low (0.342) thresholds of the RF score. In the same manner, we measured the performance of the individual encoding scheme of KSAAP, AAindex, SPIDER2, PEP2D, and pKSAAP at each level of Sp. When the same threshold values of the RF score were applied to prediction of the test dataset, the high, moderate and low levels of Sp were calculated as 0.871, 0.747, and 0.636, respectively.

To assess the performance of the PreAIP using the measures of Ac, Sp, Sn, MCC, and AUC, a 10-fold CV test was used. For the 10-fold CV, original training samples were randomly and equally picked up into 10 subclasses. Among 10 subclasses, one subclass was singled out as the test sample, and the remaining 9 subclasses were considered as the training sample. Then we computed all performance measures for each predictor. We repeated this procedure 10 times by changing the training and test samples. Eventually, we calculated the average value of each performance measure for each predictor.

## Results and Discussion

### Sequence Preference Analysis of AIPs

To investigate the amino acid preference of positive and negative AIPs, we performed sequence compositional preference analysis using the amino acids from the 1 to 15 N-terminal residues of training sets. The length of the AIPs ranged between 4 and 25 amino acid residues in this study. The average length of AIPs was 15 amino acids. Since Ialenti et al. suggested that the AIP activity is located in the N-terminal region of the molecule (Ialenti et al., 2001), we investigated the 1 to 15 N-terminal amino acids by the sequence compositional preference analysis. A non-existing residue was coded by “O” to fill the corresponding position of the AIPs.

**Figure 2:**
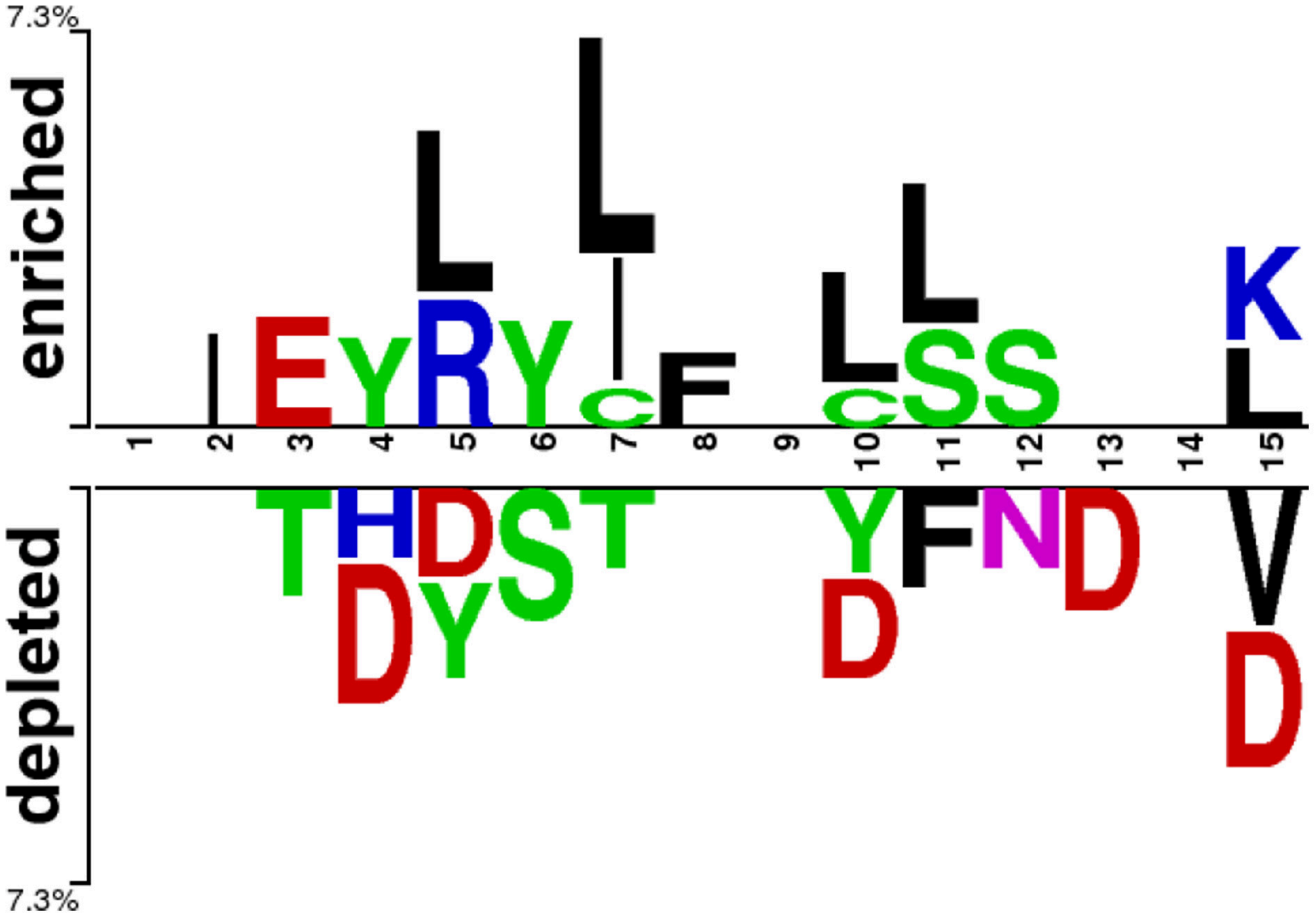
Sequence logo representation of positive and negative AIPs. The upper portion (enriched) is represented by positive AIPs, while lower portion (depleted) negative AIPs. The statistically significant local sequence within the N-terminal 15-residues of AIPs was plotted with *p* < 0.05 by Welch's *t*-test.

At first, we submitted the 1 to 15 N-terminal amino acids of positive and negative AIPs to the sample logo online server (http://www.twosamplelogo.org/) to generate the sequence logo representations (Figure 2). The height for each amino acid was in proportion to the percentage of positive (over-represented) or negative (under-represented) peptides. The logos were scaled according to their statistical significance threshold of *p* < 0.05 by Welch's *t*-test. Leucine (L) at positions 5, 7, 10, 11, and 15, cysteine (C) at position 7 and 10, isoleucine (I) at positions 2 and 7, arginine (R) at position 5, phenylalanine (F) at position 8, and lysine (K) at position 15 were significantly overrepresented compared with other amino acids, while aspartic acid (D) at positions 4, 5, 10, 13, and 15, threonine (T) at positions 3 and 7, valine (V) at position15 were significantly underrepresented. In addition, tyrosine (Y) at positions 4 and 5 was overrepresented, while Y at positions 5 and 10 underrepresented. These results suggested that positive and negative AIPs are significantly different.

**Figure 3:**
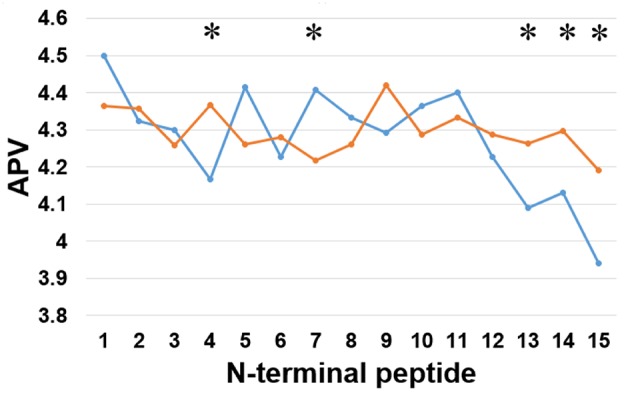
Comparison of evolutionary information of positive and negative AIPs. Blue lines represent the positive AIP, while orange lines the negative AIPs. “^*^” represents that the APV is statistically different between both the AIPs, with *p* < 0.05 by the KW test.

Secondly, we examined the evolutionary conservation features of the PreAIP using the average PSSM value (APV) for each amino acid within 1 to15 N-terminal amino acids of AIPs. The evolutionary conservation information of APV of both the positive and negative AIPs is illustrated in Figure 3. Some of amino acid positions of positive and negative AIPs showed significantly different scores. Furthermore, a nonparametric Kruskal–Wallis (KW) test was used to examine whether positive and negative AIPs were significantly dissimilar. The *p*-values were calculated and corrected by the Bonferroni test (Table S2).

**Figure 4:**
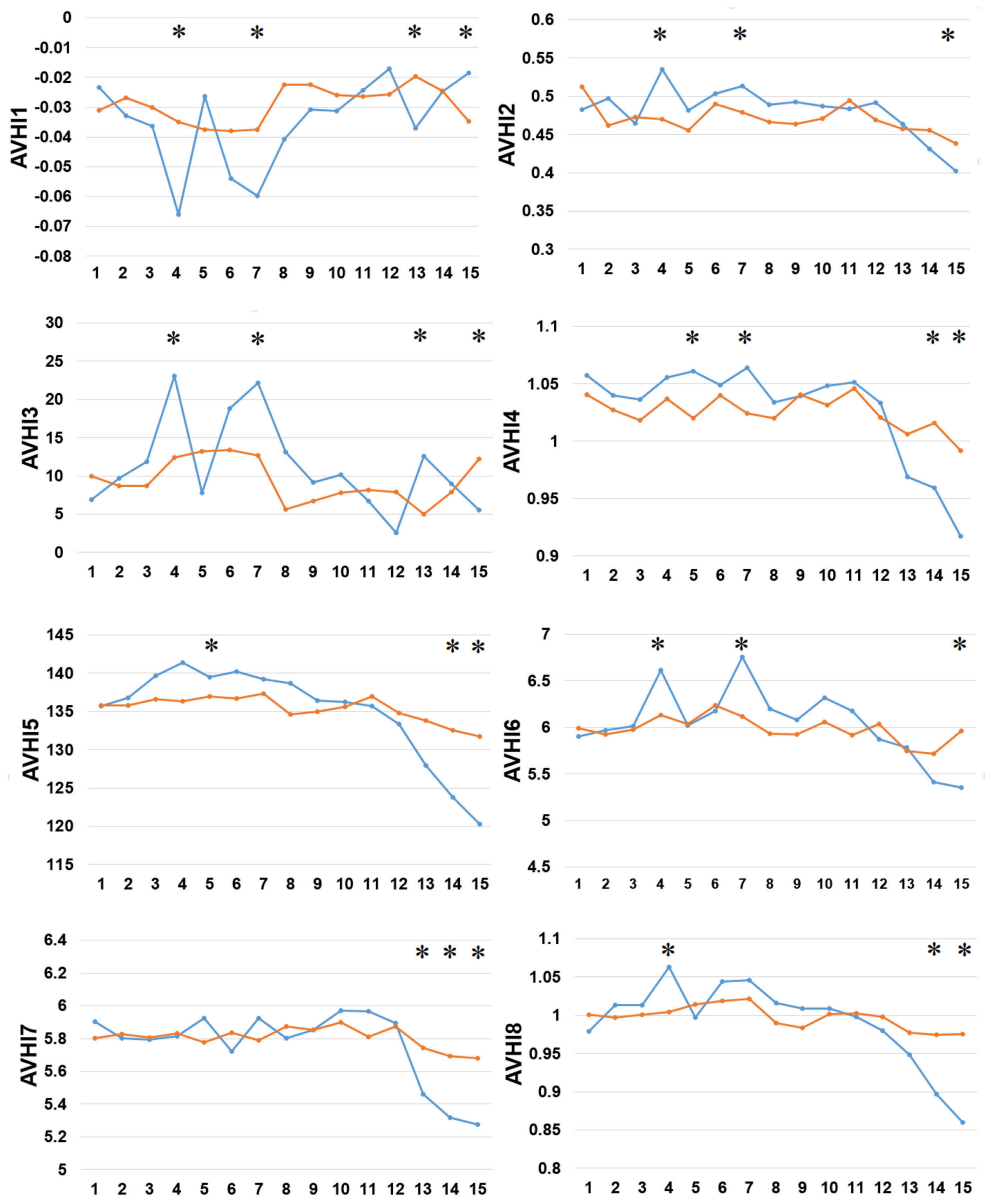
Comparison of eight high-quality amino acid indices between two samples of AIPs. The eight high-quality amino acid indices from HI1 to HI8 are placed at the centers of eight amino acid index clusters, which indicate high residue propensities of AAindex. The row represents the N-terminal peptide, while the blue lines signify the positive AIP and the orange lines the negative AIPs. “^*^” represents that the amino acid indices are statistically different between both the samples with *p* < 0.05 by the KW test.

Thirdly, we examined the AAindex encoding features of PreAIP. Eight types of informative amino acid indices were used and named HI1 to HI8 as the input feature vectors from the AAindex database. We examined these HI amino acid properties of both the positive and negative AIPs. As illustrated in Figure 4, the average values of the eight indices were renamed as AVHI1 to AVHI8. These indices represented the amino acid compositions of intracellular proteins. Some of the AIPs had distinct amino acid compositions in the eight high-quality amino acid indices between two samples of AIPs (Figure 4). The KW test was used to examine whether two samples of AIPs were significantly dissimilar with respect to the eight HI properties. The *p*-values were calculated and corrected by the Bonferroni test (Table S3). Significantly different AAindex values with *p*-value <0.05 appeared at some positions of AIPs, as marked with “^*^” in Figure 4.

**Figure 5:**
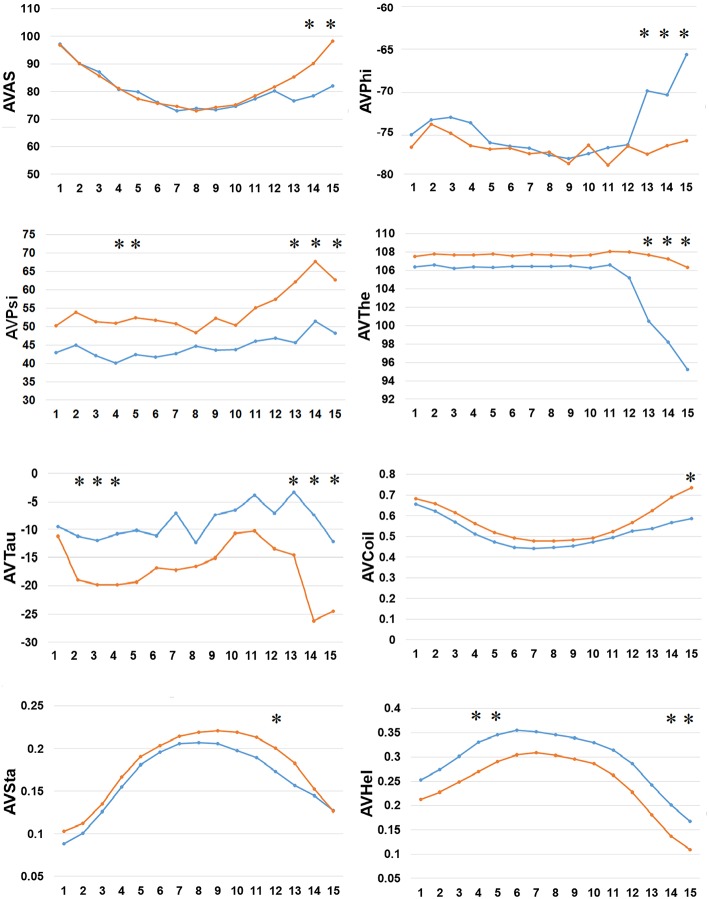
Comparison of 8 types of the SFs by SPIDER2 between positive and negative AIPs. The row represents the N-terminal peptide, while the blue lines signify the positive AIPs and the orange lines the negative AIPs. “^*^” represents that the SFs are statistically different between both the samples with *p* < 0.05 by the KW test.

Finally, we examined the difference in 8 types of SFs by SPIDER2 between the positive and negative AIPs, as shown in Figure 5. We calculated the average value of 8 types of SFs for SPIDER2: ASA, phi, psi, theta, tau, coil, stand, and helix of both the positive and negative AIPs. The average features were represented as AVAS, AVPhi, AVPsi, AVThe, AVTau, AVCoil, AVSta, and AVHel (Figure 5). We plotted these average values of SFs with respect to the 1–15 N-terminal AIPs. Distinguished differences were observed between the positive and negative samples of AIPs. The KW test was employed to examine whether two sample of AIPs were significantly dissimilar among the eight SFs. The *p*-values were calculated and corrected by the Bonferroni test (Table S4). Significantly different SFs were perceived at some positions of AIPs, with a *p*-value <0.05, as indicated with “^*^” in Figure 5.

The above analysis of residue preference between the positive and negative AIPs suggested that the combination of the primary sequence, evolutionary, and structural amino acid occurrences achieves a precise prediction.

### Overall Prediction Performance of PreAIP

The selected five descriptors (AAindex, KSAAP, SPIDER2, PEP2D, and pKSAAP) were separately used for prediction of AIPs. Optimization of multiple encoded features is generally essential in the training model to reduce dimensionality while retaining the significant feature. To achieve this, we performed multiple rounds of experiments to select appropriate feature vectors using the IG feature selection via 10-fold CV test on training set; however, it turned out that the IG feature selection did not improve prediction performance. Thus, the IG feature was used to collect significant features and for interpreting a superiority of KSAAP encoding.

**Figure 6:**
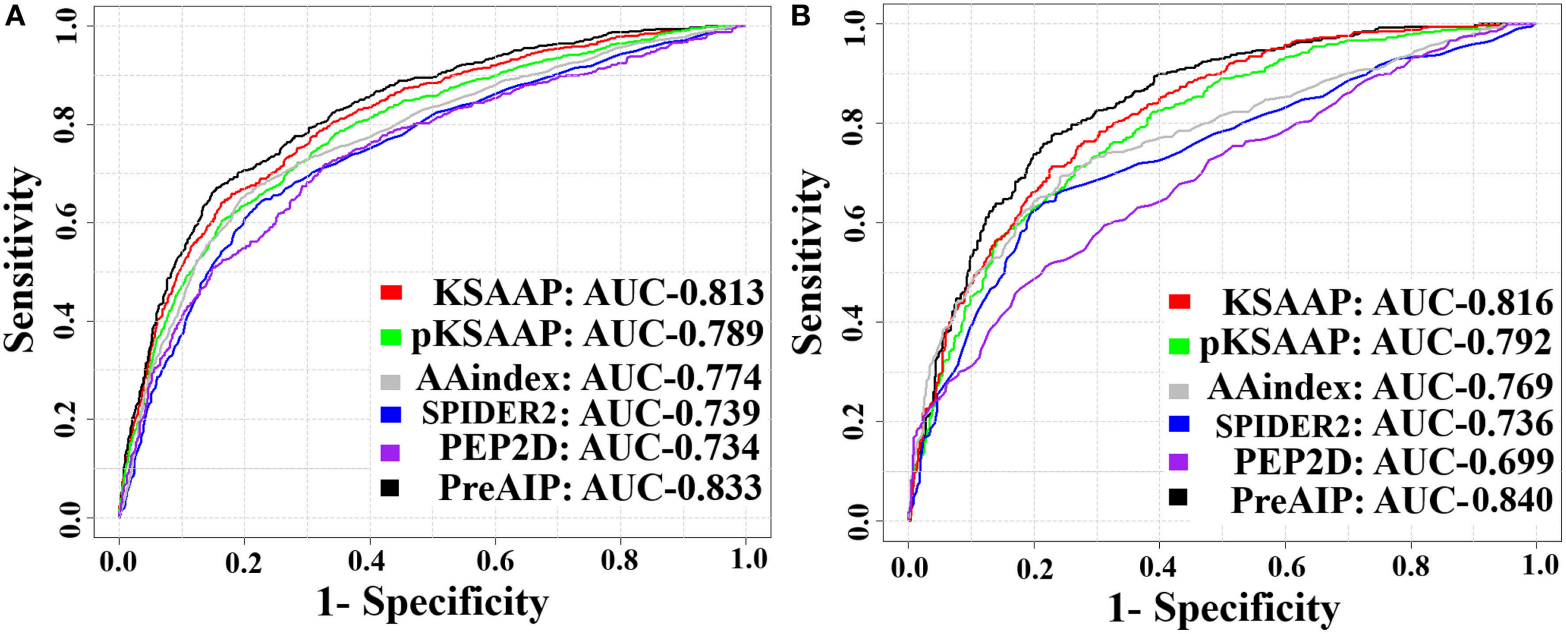
ROC curves of the various prediction models. **(A)** 10-fold CV test on a training dataset and **(B)** test dataset. The PreAIP combined the KSAAP, pKSAAP, and AAindex methods. High AUC values show accurate performance.

We accessed the performances of the training model of five successive encoding methods of AAindex, KSAAP, SPIDER2, PEP2D, and pKSAAP through a 10-fold CV test using the RF classifier. The prediction results by each of five encoding features and the “Combined features” are shown in Figure 6A. The AUCs of AAindex, KSAAP, SPIDER2, PEP2D, and pKSAAP were 0.774, 0.813, 0.739, 0.734, and 0.789, respectively. The KSAAP performed best for the 5 single encoding approaches in terms of Sn, MCC and AUC (Table 1). The “Combined features” (PreAIP) showed better performance with an AUC of 0.833 than any other single feature. It is noted that “Combined features” means a linear combination of the RF scores (Materials and Methods). Moreover, the PreAIP presented the highest AUC value (0.840) in the test dataset (Figure 6B). The performance of PreAIP was effective and reasonable for all the tested cases (Figure 6) and was best in the AIP prediction.

**Table 1:** AUC values for prediction performance of the training dataset by 10-fold CV test.

**Methods**	**Sp**	**Sn**	**Ac**	**MCC**	**AUC**	***p*-value**
pKSAAP	0.798	0.647	0.738	0.450	0.789	0.017
AAindex	0.795	0.644	0.735	0.448	0.774	0.012
SPIDER2	0.765	0.434	0.633	0.235	0.739	0.004
PEP2D	0.769	0.411	0.629	0.219	0.734	0.004
KSAAP	0.805	0.656	0.745	0.463	0.813	0.118
PreAIP^*^	0.806	0.709	0.767	0.508	0.833	

* *PreAIP is the linear combination of the RF scores estimated by SPIDER2, PEP2D, KSAAP, AAindex, and pKSAAP encoding schemes and their weight coefficients are 0.00, 0.00, 0.15, 0.25, and 0.6, respectively. A p-value was computed based on the final model of AUC values by using a Wilcoxson matched-pair signed test*.

To present the known AIPs in the training dataset, we used BLAST to search the (weak) homologs, and ranked them to obtain the best hit e-value (Bhasin and Raghava, 2004). Total 256 positive and 397 negative hits were found out of 1,258 positive and 1,887 negative samples by BLASTP with an e-value of 1.0 × 10^−2^. The reduced numbers of the samples may be due to the peptide length of 5–25. Then, we measured the BLAST performances through 10-fold CV test. The prediction performances of Sp, Sn, Ac, MCC, and AUC were 0.752, 0.269, 0.563, 0.159, and 0.632, respectively, which were lower than those by the other sequence encoding-based models. Therefore, we did not consider BLAST for final prediction.

**Figure 7:**
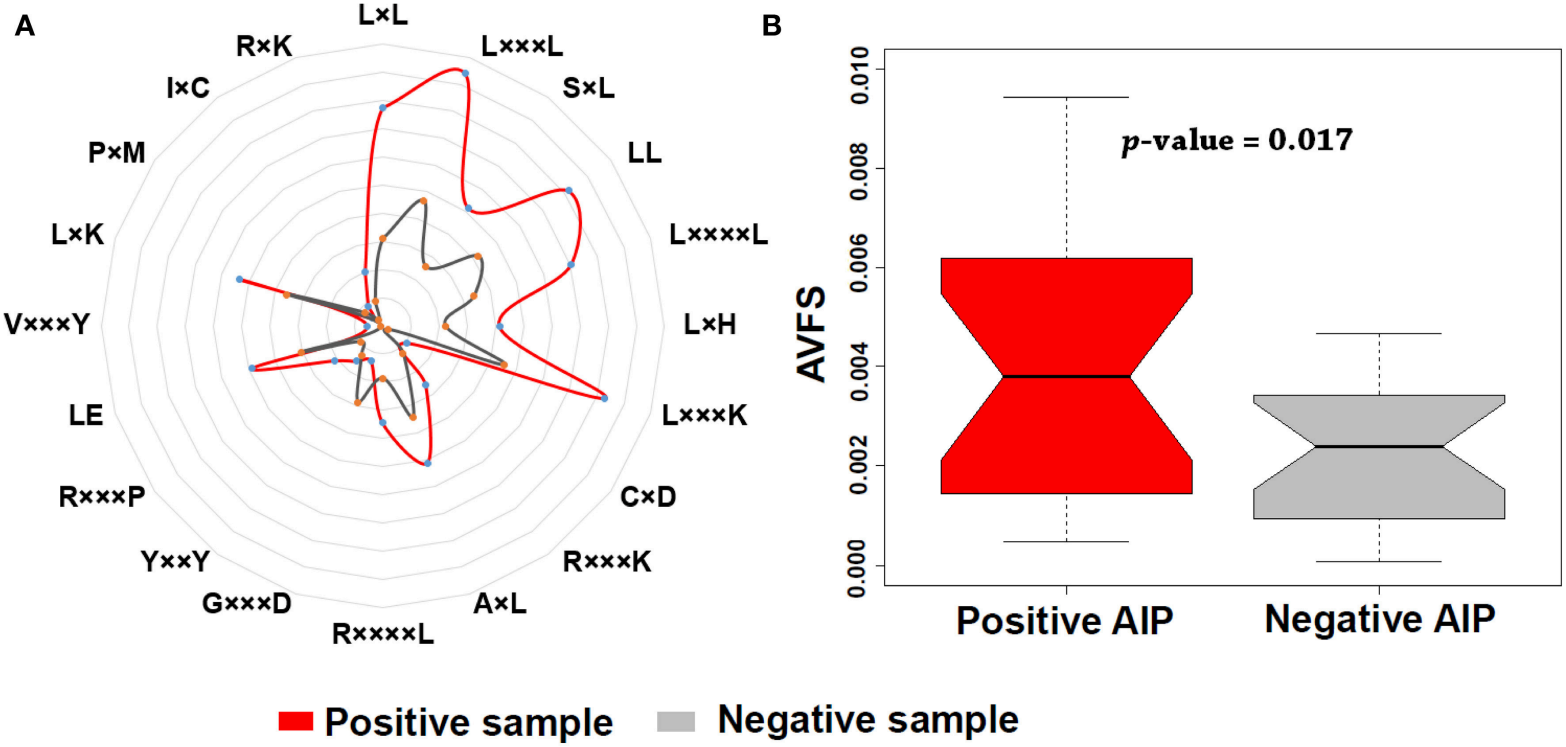
Top 20 amino acid pairs selected by the IG feature of the KSAAP method. **(A)** The radar diagram is represented by the composition of each amino acid pair whose length is proportional to the composition of KSAAP features. **(B)** Box plot shows the top 20 average value of feature scores (AVFS) by the IG. Red color denotes the positive AIPs, while gray color denotes the negative AIPs. The *p*-value is computed by two-sample *t*-test.

In addition, we found that KSAAP performed best for all the five single encoding methods. To investigate the most significant residue of the KSAAP method, the top 20 amino acid pairs of AIPs were examined through the IG feature selection. The top 20 significant residue pair scores and their corresponding positions are listed in Table S5. These significant features are also presented using a radar diagram (Figure 7A). For example, the feature sequence motif “L×L,” which is represented by 1-spaced residue pair of “LL,” is the most important residue pair, where “×” stands for any amino acid. The feature “L×××L” represented the second enriched motif surrounding positive samples of AIPs. Similarly, the feature “LL,” which represents a 0-spaced residue pair of “LL,” is important and enriched in the negative samples AIPs. Similarly, to keep other *k*-space amino acid pairs from KSAAP, the same exemplification was employed. Residue preference analysis demonstrated that “L,” “Y,” “C,” “D,” and “I” residues frequently appear for AIPs (Figures 2, 7A). These residues are expected to play a key role in the recognition of AIPs. To characterize the top 20 KSAAP-specific features, we compared the numbers of positive and negative AIPs. Figure 7B showed the top 20 average value of feature scores (AVFS) by the IG. The average of top 20 features was significantly different between two samples of AIPs with *p* < 0.05, suggesting the effectiveness of the KSAAP encoding. The significant residue pair scores are listed in Table S5, which provides some insights into the sequence patterns of the AIPs. They deserve further experimental validation.

### Comparison of PreAIP With Existing Predictors Using Test Dataset

We evaluated the performances of PreAIP along with that of existing predictors on the test dataset. We submitted the test set to the AIPpred (Manavalan et al., 2018) and AntiInflam (Gupta et al., 2017) servers to assess the performance. It is noted that AntiInflam server provides different thresholds values. We used two threshold values of −0.3 and 0.5 and renamed as less accurate (LA) and more accurate (MA) models (Gupta et al., 2017), respectively. The AIPpred represents the state-of-the-art predictor available. The average performances of the LA, MA, AIPpred, and PreAIP are illustrated in the Table 2. The LA showed the highest Sp (0.892) with the lowest Sn (0.258), MCC (0.197), and AUC (0.647) for all the predictors. The PreAIP with the high threshold presented much higher Sn (0.618) Ac (0.770), MCC (0.512), and AUC (0.840) than LA, while it provided Sp (0.871) comparable to LA. The PreAIP with the low threshold showed the highest Sn (0.863), while keeping Sp, Ac, MCC, and AUC at a high level. While the AIPpred presented considerably high values to all the measures of Sp, Sn, Ac, MCC, and AUC, the PreAIP with the moderate threshold outperformed the AIPpred, presenting well-balanced, high prediction performances. The PreAIP performance improvement was found distinct on the test dataset by the Wilcoxson matched-pair signed test, demonstrating its ability to predict unseen peptides.

**Table 2:** Performance comparison with exiting predictors using test dataset.

**Predictor**	**Threshold**	**Sp**	**Sn**	**Ac**	**MCC**	**AUC**	***p*-value**
AntiInflam (LA)	−0.3	0.892	0.258	0.638	0.197	0.647	<0.001
AntiInflam (MA)	0.5	0.417	0.786	0.565	0.210	0.706	<0.001
AIPpred	Server	0.746	0.741	0.744	0.479	0.813	0.039
PreAIP	High	0.871	0.618	0.770	0.512	0.840	
	Moderate	0.747	0.784	0.762	0.522	0.840	
	Low	0.636	0.863	0.727	0.492	0.840	

*A p-value was computed based on AUC values by using a Wilcoxson matched-pair signed test and p < 0.05 indicates a statistically significant difference between the proposed PreAIP and each selected method. The performances of AntiInflam LA and MA methods were computed using default threshold (server) values of −0.3 and 0.5, respectively. The AIPpred threshold was the same as given by its server*.

### Comparison of PreAIP With AIPpred Using Training Dataset

We compared the performance of the proposed PreAIP with the AIPpred using the same training dataset. In this study, the same dataset as the AIPpred set was used to make a fair comparison for prediction performance of AIPs. As shown in Table 3, the PreAIP achieved a better performance than the AIPpred in terms of Ac, Sp, Sn, MCC, and AUC. The AUC value was nearly 3% higher than the AIPpred predictor. The PreAIP performance (AUC) improvement over the AIPpred was demonstrated on the training set by the Wilcoxson matched-pair signed test (Table 3).

**Table 3:** Performance comparison of PreAIP with AIPpred using training dataset.

**Methods**	**Threshold**	**Sp**	**Sn**	**Ac**	**MCC**	**AUC**	***p*-value**
AIPpred	Default given in the server	0.711	0.758	0.730	0.460	0.801	0.034
PreAIP	High	0.903	0.632	0.795	0.566	0.833	
	Moderate	0.801	0.719	0.768	0.520	0.833	
	Low	0.709	0.784	0.739	0.484	0.833	

*A p-value was computed based on AUC values by using a Wilcoxson matched-pair signed test and p < 0.05 indicates a statistically significant difference between the proposed PreAIP and AIPpred*.

### Comparison of Different Machine Learning Algorithms

The performance of the RF was compared to the three widely used machine learning algorithms, NB, SVM, and ANN by using the same training datasets and features, as shown in Table 4. The AUC values of the prediction by the five algorithms were calculated by a 10-fold CV test, while using the SPIDER2, PEP2D, AAindex, KSAAP, and pKSAAP encodings and their combined method. The RF provided higher AUC than any other algorithms for all the encoding methods and their combined method.

**Table 4:** AUC values of AIP prediction by different machine learning algorithms based on a 10-fold CV test.

**Algorithms**	**SPIDER2**	**PEP2D**	**AAindex**	**KSAAP**	**pKSAAP**	**Combined**
RF	0.739	0.734	0.774	0.813	0.789	0.833
NB	0.659	0.655	0.707	0.729	0.717	0.736
SVM	0.698	0.677	0.738	0.766	0.749	0.779
ANN	0.662	0.649	0.716	0.741	0.736	0.753

“*Combined” indicates that the performance of the optimized combined features. The combined score of RF was given as the sum of the five SPIDER2, PEP2D, AAindex, KSAAP, and pKSAAP features with weight values of 0.00, 0.00, 0.15, 0.25, and 0.6 respectively. In the same way, the weight values of NB, SVM, and ANN were given as (0.00, 0.00, 0.10, 0.35, and 0.55), (0.00, 0.00, 0.22, 0.45, and 0.33), and (0.00, 0.00, 0.18, 0.5, and 0.32), respectively*.

### The Effect of Peptide Redundancy on the Predictive Model

The peptide redundancy may lead to the overestimation on the predictive performance. Therefore, we performed the CD-HIT with 60% identity cutoff at the peptide level (Huang et al., 2010). After removing the 60% sequence redundancy, we re-assembled a training dataset that contained 1,098 positive and 1,226 negative samples, and the test dataset that contained 308 positive and 275 negative samples. While the overall performance (AUC = 0.821) of the PreAIP by the 10-fold CV test decreased slightly (Table S6), the PreAIP could still achieve the best performance on the independent testing dataset (Figure S1). The PreAIP achieved 6 and 8% higher AUC values than the AntiInflam and the AIPpred, respectively, demonstrating that the PreAIP with the 60% peptide redundancy removal provides a stable or competitive performance compared with the other predictors, as well as the 80% peptide redundancy removal.

### Advantages of PreAIP

In theoretical viewpoints, comparison of the proposed PreAIP with existing predictors is summarized: (1) The PreAIP investigated the primary sequence, physicochemical properties, structural, and evolutionary features, although the AIPpred and AntiInflam predictors used only primary sequence encoding method. For instance, in AntiInflam method (Gupta et al., 2017), studied hybrid features based on primary sequence encoding schemes such as amino acid composition (AAC), dipeptide composition (DPC), and tripeptide composition with SVM algorithm. The AIPpred (Manavalan et al., 2018) studied individual composition (AAC, AAindex, DPC, and chain-transition-composition) through multiple machine learning algorithms. (2) Since existing prediction tools did not control the Sp level, users cannot understand which AIP is highly positive or negative from their servers. On the other hand, the PreAIP controlled Sp at high, moderate and low levels by changing the threshold of the RF scores, based on 10-fold CV test results. A limitation of the PreAIP is that the employed dataset is still small, but we believe that the dataset will grow to enable intensive identification of AIPs. In addition, the calculation speed remains to be improved. The processing time of the PreAIP was <3 min for one peptide sequence, where the generation of PSSM profiles requires a long time.

### Server of PreAIP

A web server of the PreAIP has been developed and publically accessible at http://kurata14.bio.kyutech.ac.jp/PreAIP/. The web application was implemented by programming languages of Java scripts, Perl, R, CGI scripts, PHP, and HTML. After submitting a query sequence to the server, it generates consecutive feature vectors. Then, the server optimizes the performances through RFs. After completing the submission job, the server returns the result in the output webpage which consists of the job ID and probability scores of the predicted AIPs in a tabular form. A user gets a job ID like “2018032900067” and can save this ID for a future query. The server stores this job ID for one month. The input peptide sequence must be in the FASTA format. Each of the 20 types of standard amino acids must be written as one uppercase letter. See the test example on the server. The length of AIP sequence was limited from 1 to 25. If users submit 200 amino acids, the PreAIP takes first 1–25 residues to analyze. When the peptide contains less than 25 residues, the PreAIP provides gaps (–) to the missing residues to compensate a peptide length of 25.

## Conclusions

We have designed an accurate and efficient computational predictor for identifying potential AIPs. It outperforms the existing methods and is effective in understanding some mechanisms of AIP identification. An IG-based feature selection method was carried out to suggest sequence motifs of AIPs from KSAAP encoding. A user-friendly web-server was developed and freely available for academic users.

## Author Contributions

MK, MH, and HK conceived and designed the study. MK and MH collected data and performed the analyses. MH, MK, and HK wrote the manuscript. All authors discussed the prediction results and commented on the manuscript.

### Conflict of Interest Statement

The authors declare that the research was conducted in the absence of any commercial or financial relationships that could be construed as a potential conflict of interest.
