# Supplementary material for: PreAIP: Computational Prediction of Anti-inflammatory Peptides by Integrating Multiple Complementary Features
Source: Front Genet. 2019 Mar 5;10:129. doi: 10.3389/fgene.2019.00129 (PMC6411759; doi:10.3389/fgene.2019.00129)
Supplement: Supplementary file 1 [file Data_Sheet_1.PDF]

PreAIP: Prediction of Anti-inflammatory Peptides by Integrating Multiple Complementary Features

Mst. Shamim Khatun[1#], Md. Mehedi Hasan[1#] and Hiroyuki Kurata[1,2*]

[1]Department of Bioscience and Bioinformatics, Kyushu Institute of Technology, 680-4 Kawazu, Iizuka, Fukuoka 820-8502, Japan.

[2]Biomedical Informatics R&D Center, Kyushu Institute of Technology, 680-4 Kawazu, Iizuka, Fukuoka 820-8502, Japan.

Table S1. Eight types of high index (HI) of AAindex properties used in this study.

AAindex ID	Index name	Properties Describtion
MIYS990104	HI1	Optimized relative partition energies
BLAM930101	HI2	Alpha helix propensity of position 44 in T4 lysozyme
BIOV880101	HI3	Information value for accessibility
MAXF760101	HI4	Alpha and turn propensities
TSAJ990101	HI5	Volumes including the crystallographic waters using standard radii and volumes.
NAKH920108	HI6	Amino acid composition of MEM of multi-spanning proteins
CEDJ970104	HI7	Amino acid composition and cellular location in proteins.
LIFS790101	HI8	Conformational preference for all beta-strands

Table S2 Statistical difference in the APVs between the positive and negative AIPs. The *p*-values were calculated using the KW test and corrected by the Bonferroni test. '*' represents *p*-values < 0.05.

N-terminal positive	*p*-value
1	5.41E-01
2	1.00
3	1.00
4	1.01E-02*
5	9.64E-01
6	1.00
7	4.64E-02*
8	1.00
9	1.00
10	1.00
11	1.00
12	1.00
13	3.64E-02*
14	2.11E-02*
15	1.79E-02*

Table S3 Statistical difference in the high index of AAindex properties between the positive and negative AIPs. The *p*-values were calculated using the KW test and corrected by the Bonferroni test. '*' represents *p*-values < 0.05.

N-terminal positive	AVHI1	AVHI2	AVHI3	AVHI4	AVHI5	AVHI6	AVHI7	AVHI8
1	1.00	1.00	1.00	1.00	1.00	1.00	1.00	1.00
2	1.00	1.00	1.00	1.00	1.00	1.00	1.00	1.00
3	1.00	1.00	1.00	1.00	1.00	1.00	1.00	1.00
4	2.91E-02*	1.15E-03*	1.01E-02*	1.00	1.00	4.01E-02*	1.00	3.01E-02*
5	1.00	1.00	5.98E-01	2.69E-02*	3.64E-02*	1.00	1.00	1.00
6	1.00	1.00	1.00	1.00	1.00	1.00	1.00	1.00
7	2.45E-02*	3.86E-02*	4.64E-02*	2.39E-02*	1.00	3.37E-02*	1.00	1.00
8	1.00	1.00	1.00	1.00	1.00	1.00	1.00	1.00
9	1.00	1.00	1.00	1.00	1.00	1.00	1.00	1.00
10	1.00	1.00	1.00	1.00	1.00	1.00	1.00	1.00
11	1.00	1.00	1.00	1.00	1.00	1.00	1.00	1.00
12	1.00	1.00	1.00	1.00	1.00	1.00	1.00	1.00
13	1.99E-02*	1.00	4.87E-02*	1.00	1.00	1.00	4.07E-02*	1.00
14	1.00	1.00	1.00	2.19E-02*	3.01E-02*	1.00	3.01E-02*	3.99E-02*
15	4.65E-02*	3.65E-02*	4.08E-02*	3.79E-02*	1.29E-02*	2.11E-02*	1.33E-02*	2.38E-02

Table S4 Statistical difference in the 8 types of SFs by SPIDER2 between the positive and negative AIPs. The *p*-values were calculated using the KW test and corrected by the Bonferroni test. '*' represents *p* values < 0.05.

N-terminal positive	AAS	Phi	Psi	The	Tau	Coil	Stand	Helix
1	1.00	1.00	3.78E-01	1.00	7.78E-01	1.00	1.00	7.08E-01
2	1.00	1.00	8.67E-02	1.00	4.67E-02*	1.00	1.00	6.67E-01
3	1.00	1.00	1.69E-01	1.00	3.69E-02*	1.00	1.00	6.54E-02
4	1.00	1.00	1.91E-02*	1.00	3.11E-02*	1.00	1.00	4.14E-02*
5	1.00	1.00	4.98E-02*	1.00	5.98E-02	1.00	1.00	4.08E-02*
6	1.00	1.00	8.88E-02	1.00	1.00	1.00	1.00	1.00
7	1.00	1.00	1.00	1.00	1.00	1.00	1.00	1.00
8	1.00	1.00	1.00	1.00	1.00	1.00	1.00	1.00
9	1.00	1.00	1.00	1.00	1.00	1.00	1.00	1.00
10	1.00	1.00	1.00	1.00	1.00	1.00	1.00	1.00
11	1.00	1.00	1.00	1.00	1.00	1.00	1.00	1.00
12	1.00	1.00	1.00	1.00	1.00	1.00	4.98E-02*	1.00
13	5.99E-02	3.75E-02*	2.87E-02*	2.87E-02*	3.67E-02*	1.00	6.56E-01	7.67E-02
14	4.40E-02*	3.13E-02*	1.00E-02*	4.13E-03*	2.97E-03*	1.00	1.00	4.97E-02*
15	3.78E-02*	2.39E-02*	1.08E-03*	1.39E-03*	1.65E-03*	4.53E-02*	1.00	1.99E-02*

Table S5 Top 20 IG features of KSAAP encoding with corresponding amino acid pair positions.

Ser. No	IG features	Amino acid pairs
1	0.01145	L×L
2	0.01077	L×××L
3	0.00909	S×L
4	0.00807	LL
5	0.00562	L××××L
6	0.00475	L×H
7	0.00446	L×××K
8	0.0044	C×D
9	0.0041	R×××K
10	0.00368	A×L
11	0.00368	R××××L
12	0.00356	G×××D
13	0.00346	Y××Y
14	0.00345	R×××P
15	0.00332	LE
16	0.00331	V×××Y
17	0.0033	L×K
18	0.0033	P×M
19	0.00328	I×C
20	0.00327	R×K

Table S6. AUC values with 60% peptide redundancy on the training dataset by 10-fold CV test

Methods	Sp	Sn	Ac	MCC	AUC
pKSAAP	0.802	0.627	0.719	0.413	0.768
AAindex	0.786	0.613	0.704	0.388	0.753
SPIDER2	0.755	0.414	0.594	0.235	0.739
PEP2D	0.761	0.365	0.574	0.199	0.693
KSAAP	0.801	0.652	0.731	0.443	0.806
PreAIP*	0.806	0.709	0.761	0.486	0.821

* PreAIP is the linear combination of the RF scores estimated by SPIDER2, PEP2D, KSAAP, AAindex and pKSAAP encoding schemes and their weight coefficients are 0.00, 0.00, 0.10, 0.35, and 0.55, respectively.

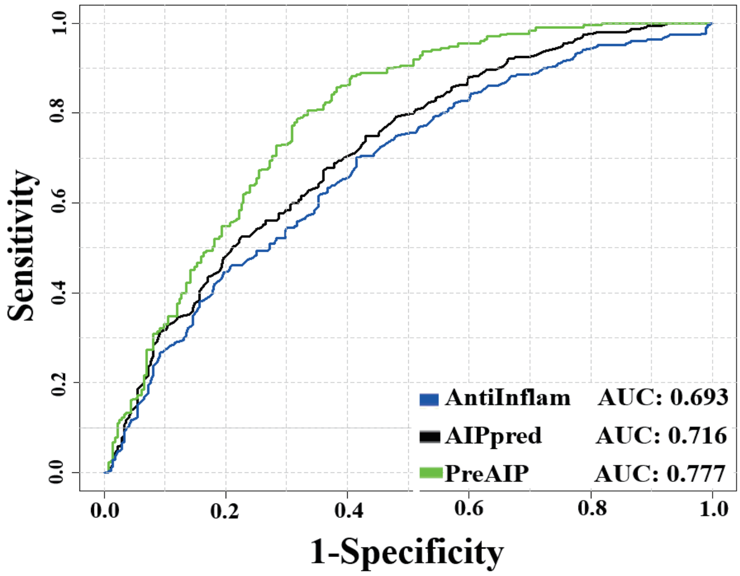

Figure S1: AUC values with 60% peptide redundancy removal on the test dataset.
